# Supplementary material for: Charge Transport across Proteins inside Proteins: Tunneling across Encapsulin Protein Cages and the Effect of Cargo Proteins
Source: Biomolecules. 2023 Jan 13;13(1):174. doi: 10.3390/biom13010174 (PMC9855946; doi:10.3390/biom13010174)
Supplement: Supplementary file 1 [file biomolecules-13-00174-s001.zip › biomolecules-2140102-supplementary.pdf]

Supplementary Information for

Charge Transport across Proteins Inside Proteins: Tunneling across Encapsulin Protein Cages and the Effect of Cargo Proteins

Riccardo Zinelli [1,2,#], Saurabh Soni [1,#], Jeroen J. L. M. Cornelissen [2], Sandra Michel-Souzy [2,], Christian A. Nijhuis [1,*]*

[1] Hybrid Materials for Opto-Electronics Group, Department of Molecules and Materials, MESA+ Institute for Nanotechnology, Molecules Center and Center for Brain-Inspired Nano Systems, Faculty of Science and Technology, University of Twente, P.O. Box 2017, 7500 AE Enschede, The Netherlands

[2] Biomolecular NanoTechnology, Department of Molecules and Materials, MESA+ Institute for Nanotechnology, Molecules Center and Center for Brain-Inspired Nano Systems, Faculty of Science and Technology, University of Twente, P.O. Box 2017, 7500 AE Enschede, The Netherlands

* Authors to whom correspondence should be addressed: c.a.nijhuis@utwente.nl (C.A.N.), s.s.m.c.michel@utwente.nl (S.M.)

[#]These authors contributed equally to this work

Summary

S1 Protein Production 3

S2 Protein Purification 4

Figure S1.......... 6

Figure S2.......... 7

Figure S3.......... 8

S3 SAM preparation. 9

S4 SAM characterisation. 10

S4.1 AFM. 10

Figure S4.......... 11

Figure S5.. 12

Figure S6.......... 12

Figure S7.......... 13

Figure S8.......... 13

Figure S9.......... 14

S1 Protein Production

A pre-culture was prepared by starting either from colonies cultured on a Petri dish or from cells preserved at -80 °C in a culture tube of Lennox Broth (LB) with the appropriate antibiotic (ampicillin 50 μg/ml, streptomycin 30 μg/ml and kanamycin 30 μg/ml depending on the plasmid used) and incubated overnight at 37 °C and 200 rpm agitation. 25 ml of pre-culture was made for each liter of final culture. The optimal starting optical density at 600 nm for Escherichia coli culturing was between 0.05 and 0.1. To obtain the proper dilution of the pre-culture and to check the growth, the optical density was measured. This was done by diluting 100 μl of pre-culture with 900 μl of milliQ water in a 1 ml cuvette that was then measured against a cuvette filled with milliQ water as a reference. The culturing was done in 5 liter Erlenmeyer flask. To 1 liter of culture, the appropriate amount of antibiotics and the calculated amount of pre-culture were added. The incubation was done at 37°C and 200 rpm agitation. The optical density was checked periodically until the optimal value to induce the gene expression between 0.4 and 0.6 was reach. The induction was done by adding 100μM or 500μM of IPTG (Isopropyl β-d-1-thiogalactopyranoside). The incubation was continued overnight at 25 °C 200 rpm agitation [1].

S2 Protein Purification

The cell cultures were centrifuged at 4000 g for ten minutes at 4 °C. The supernatant was discarded and the cell pellets were collected and re-suspended in a lysis buffer. 10 ml of this buffer was prepared for a liter of culture using the following recipe:

a. Hepes 50 mM (pH 8);
b. NaCl 150 mM;
c. 2-mercaptoethanol 15 mM;
d. Imidazole 30 mM (only for His-tag purified proteins);
e. EDTA 1 mM;
f. MgCl2 20 mM;
g. Protease inhibitor 1 mM;
h. RNAse 30 µg/ml;
i. DNAse 20 µg/ml;
j. Lysozyme 0.5 mg/ml.

The re-suspended cells in lysis buffer were then transferred into a 50 ml Greiner tube, placed in ice and sonicated twice for 1 min at 80% amplitude with 3 s pulse (Fisherbrand ultrasonic processor 120 W, 20 kHz). The lysis buffer was allowed to sit in contact with the cells for at least 10 minutes. The Greiner tube containing the lysed cells was centrifuged at 4000 g for 10 minutes at 4 °C. The supernatant was collected and transferred into ultracentrifuge tubes and centrifuged at 140000 g for 40 min at 4 °C. The supernatant was carefully collected (30 µl "loading" were stored in the fridge for future characterization through SDS-PAGE) and further purified by using an FPLC machine (BioRad NGC™ chromatography system). The solution obtained was poured into a super-loop and loaded into an affinity column (StrepTrapTM HP

5 ml for the C-strep tagged proteins, or His-Trap 5 ml for the His10 tagged proteins) with a 1 ml/min flow. At the end of the loading, 30 µl were collected as "flow-through" and stored in a 1.5 ml Eppendorf in the fridge for a future SDS-PAGE). The column was then washed with a washing buffer. The buffer was made following the recipe: 50 mM Hepes, 150 mM NaCl, 0.1% 2-mercaptoethanol, pH 8 and 30 mM Imidazole (only for His10-Tagged proteins). At the end of the washing step, 30 µl "washing" were collected in a 1.5 ml Eppendorf in the fridge for SDS-PAGE analysis). The Product was eluted with 15 ml of eluting buffer: 50 mM Hepes, 150 mM NaCl, 0.1% 2-mercaptoethanol, pH 8 and either 5 mM desthiobiotin (for C-strep tagged proteins) or 500 mM imidazole (for His10-tagged proteins), fractions of 1 ml were collected.

To complete the purification, by separation, based on the size of eventually aggregated protein, monomers and other impurities, a final size exclusion chromatography step was performed in a Superose® 6 Increase 10/300 GL column. The sample, which consists usually of one or two fractions of 1 ml each, is loaded in an appropriate capacity loop. It is also possible to concentrate together two fractions by centrifugation steps in Amicon Ultrafilter unit 100kDa (15 ml), at 2000 rpm for 2 min, until the volume is reduced to 1 ml. It is important to re-suspend the solution after every centrifugation step and to stop the concentration if the solution starts being cloudy, a clear sign of aggregation and precipitation of the encapsulin. The sample is injected into the column and eluted at 0.5 ml/min (maximum pressure 4.00 MPa, maximum with the following buffer: 50 mM Hepes, 150 NaCl, 0.1% 2-mercaptoethanol and pH 8. The peak for encapsulin cages is around 12.50 ml of eluted volume. Before this peak, another peak of aggregated cages can be sometimes found, at 9 to 12 ml of eluted volume.

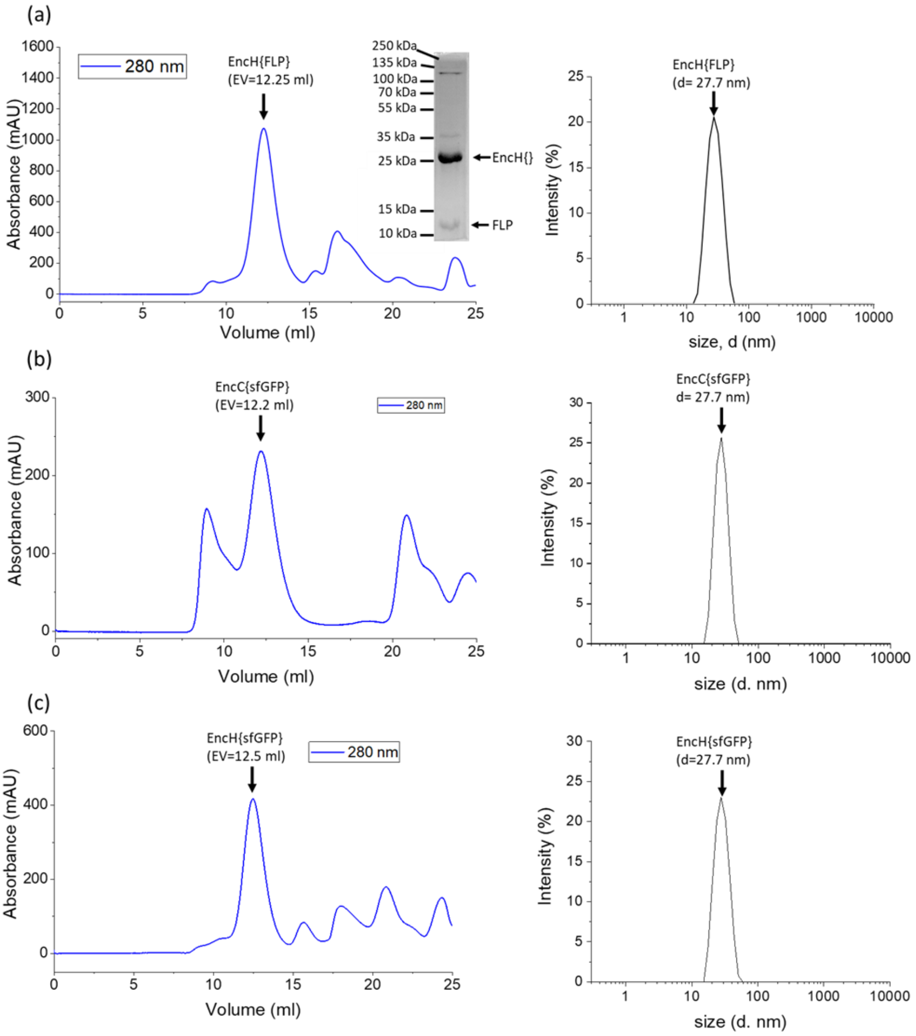

Figure S1. (a) Size exclusion chromatogram of EncH{FLP} showing the elution peak of the encapsulin cage centered at an elution volume of 12.25 ml. on the left it is reported the SDS-PAGE showing an intense band associated with the encapsulin monomer between the 25kDa and 35 kDa. The DLS graph shows a monodisperse distribution with an average diameter of 27.7 nm. (b) Size exclusion chromatogram of EncC{sfGFP} showing the elution peak of the encapsulin cage centered at an elution volume of 12.2 ml. The DLS graph shows a monodisperse distribution with an average diameter of 27.7 nm. (c) Size exclusion chromatogram of EncH{sfGFP} showing the elution peak of the encapsulin cage centered at an elution volume of 12.5 ml. The DLS graph shows a monodisperse distribution with an average diameter of 27.7 nm.

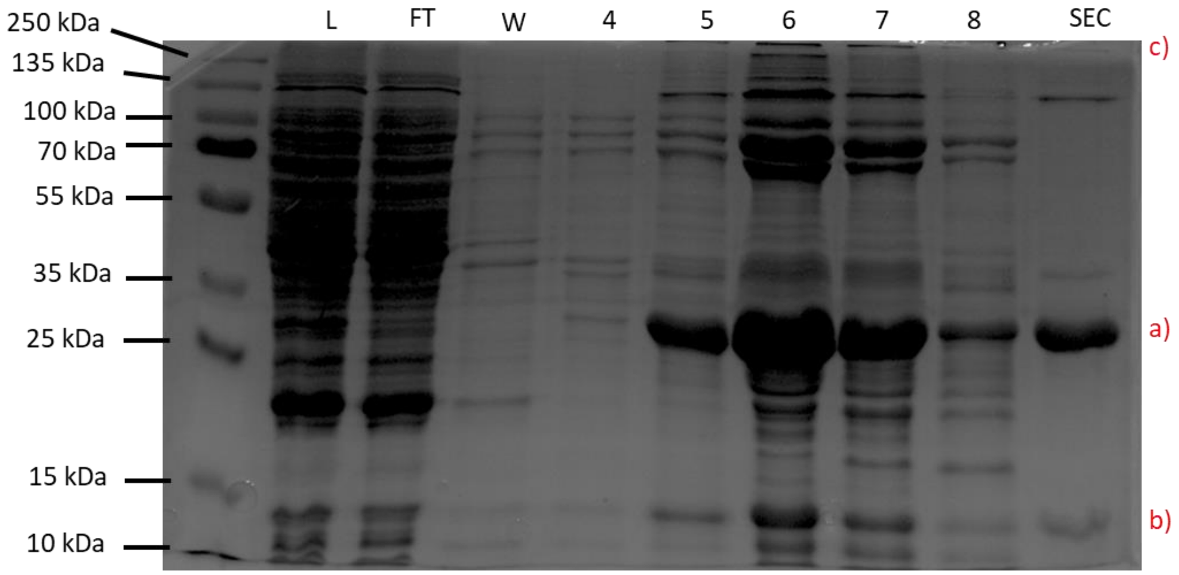

Figure S2. SDS-PAGE gel of EncH{FLP}. L=loading fraction, FT=flow-through fraction, W=washing fraction. Fraction 4, 5, 6, 7 and 8 from the affinity chromatography. Fraction 6 is the most concentrated but there is still a high concentration of impurities. However, it is possible to see that the encapsulin results in increased concentration compared to the loading and the flow-through. SEC = size exclusion chromatography. It is possible to distinguish three main lines: a) encapsulin monomer while b) could correspond to FLP monomer, and c) is compatible to aggregated and non-digested material, less clearly visible.

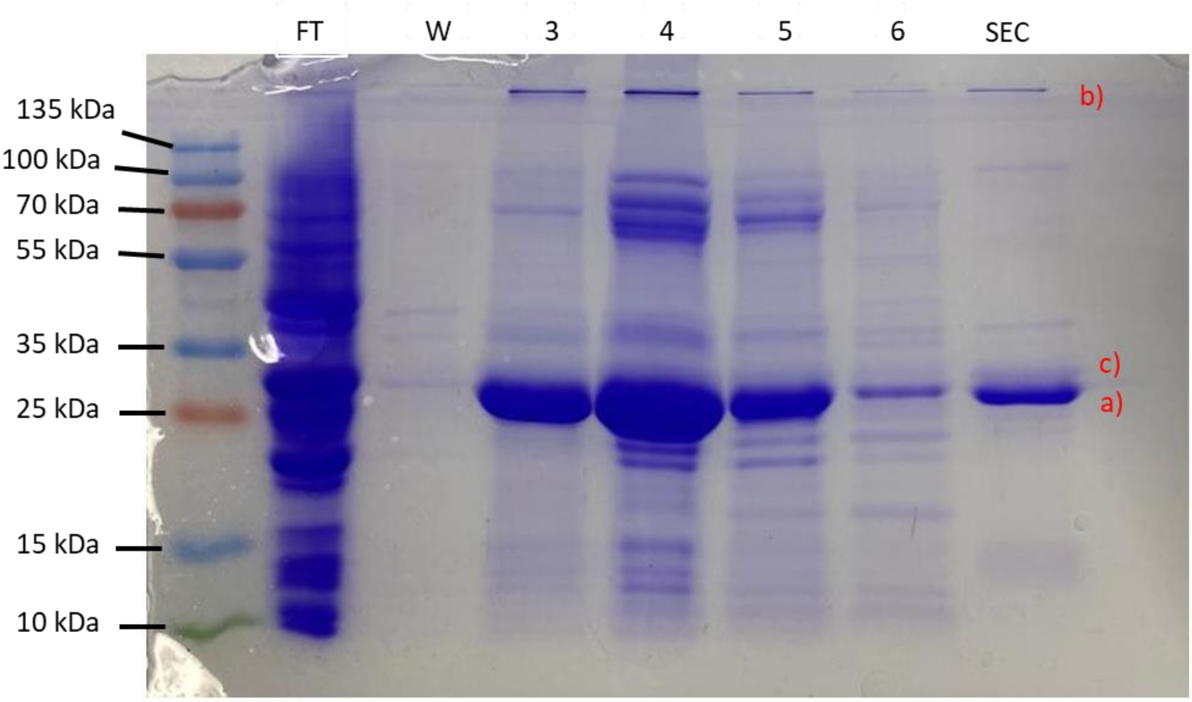

Figure S3. SDS-PAGE gel of EncH{sfGFP}. FT = flow-through, W =washing, 3-6 correspond to the fractions eluted during the affinity purification. SEC = final product of the size exclusion purification. a) Line associated with the encapsulin monomer, b) aggregates and non-digested encapsulin cage, and c) sfGFP line.

S3 SAM preparation.

Prewashed glass substrates were used for EGaIn and AFM to prepare SAMs via established procedure [2]–[4]. A 3 mM ethanolic solution of lipoic acid was prepared. 3 ml of solution were used for each sample. Degassed absolute ethanol and lipoic acid, flushed with nitrogen, were transferred into a glove box and mixed to prepare a 3 mM solution. Owing to the rectangular geometry of the EGaIn substrates, they could be immersed with the Au side facing down in a glass vial, avoiding aggregation and precipitation on the surface. The Au substrates were immersed in the solution for 2 hours and then rinsed with absolute ethanol (about 2 ml). A degassed ethanolic solution containing EDC (75 mM) and NHS (50 mM) was prepared inside a glovebox. 3 ml were used for each sample. The rinsed substrates with lipoic acid SAMs from the previous steps were immersed in the solution for 2 h at room temperature. Then the substrates were rinsed with absolute ethanol (about 2 ml). A 0.10 mg/ml protein in buffer solution was prepared. For each sample at least 1.2 ml per sample was used. The activated substrates were immersed in the protein solution for 2 h at 4 °C and then rinsed with fresh buffer and water. The SAMs were dried gently under a nitrogen flow.

S4 SAM characterisation.

S4.1 AFM.

Scans of 1 × 1 μm were also taken from the SAMs that were studied using the EGaIn technique, to characterize the surface roughness of the SAMs studied and the encapsulin cages. Figure S5a shows the roughness of the bare Au substrate is very low comparable to reported values in the literature [5], [6]. A significant difference can already be seen for the lipoic acid SAMs, in which the roughness increases slightly and aggregated particles of a few nanometers appear on the surface. The roughness and aggregation on the surface further increase with the EDC-NHS activation of the SAM's surface. Even though some particles seems to appear on the surface already at this early stage, the final structure of the encapsulin functionalized SAMs is completely different, as it is characterized by a higher roughness and evident as the proteins have a diameter of 25-30 nm, as shown in figure S5d-g. Depending on the cargo contained in the encapsulin nanocompartment, the height profile changes significantly, with an increase in height going from the encapsulin without cargo EncH{}, in Figure S5d, to EncH{sfGFP} in Figure S5e, in which the height slightly increases. A dramatic difference arises in the comparison of the previously mentioned variants with EncH{FLP}, shown in Figure S5f-g, that shows well defined particles of 8-12 nm in height, both in the packed SAMs and in the diluted sample, where the encapsulin cages are sparsely distributed on the Au [7].

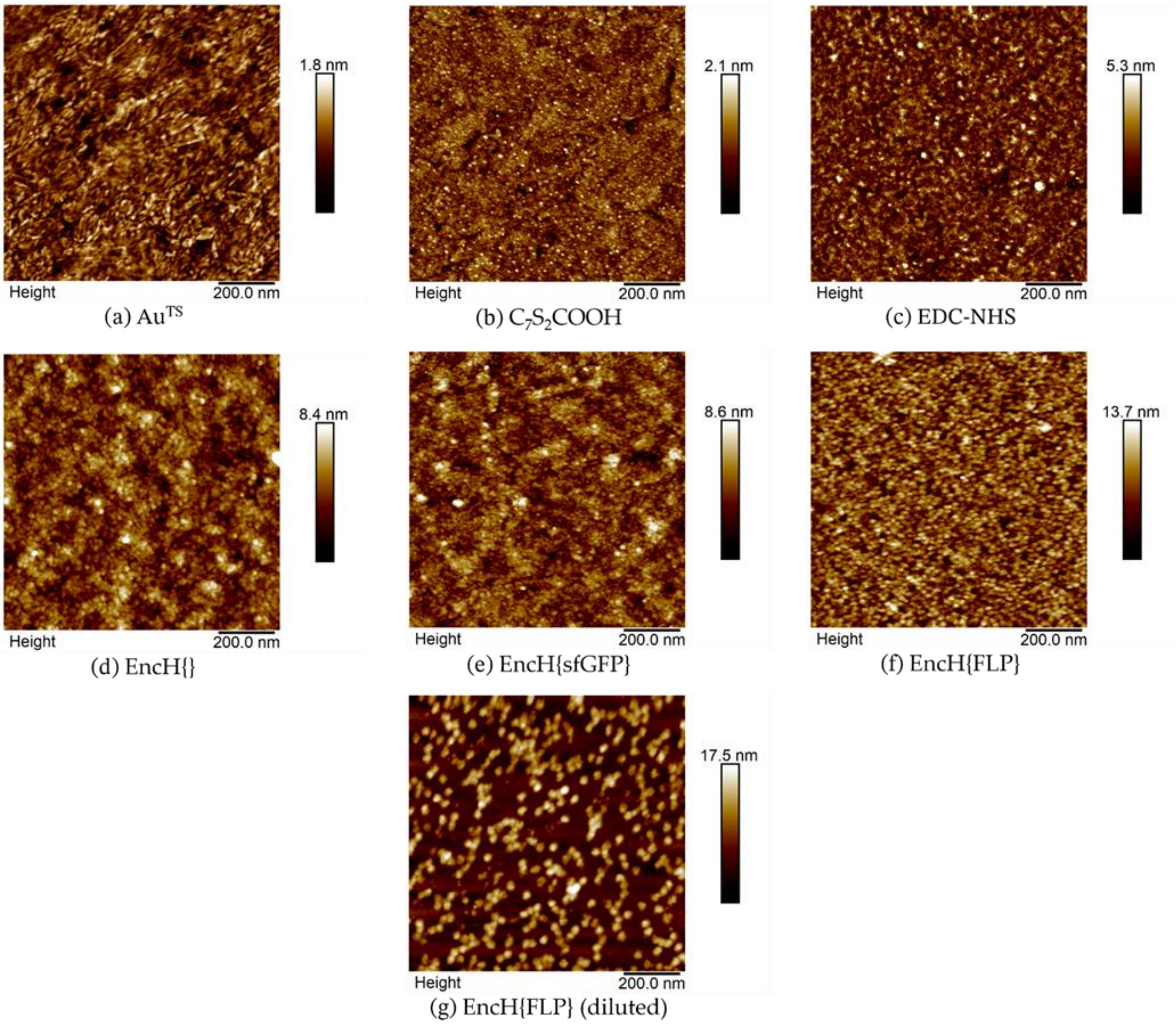

Figure S4. Surface characterization of the SAMs using AFM. (**a**) Au substrate with no functionalization on the surface. The following pictures are of the SAMs with (**b**) C_7S_2COOH, (**c**) EDC-NHS activated lipoic acid SAMs, (**d**) EncH{} without cargo, (**e**) EncH{sfGFP}, (**f**) EncH{FLP}, and (**g**) diluted SAM of EncH{FLP} showing individual protein particles on the surface.

S5. EGaIn Current-Voltage Measurements.

Along with the encapsulin samples, SAMs of Horse spleen apoferritin (HS-apoferritin) were also produced as an internal reference (and to compare with existing studies on ferritin-based cages [4], [7]) and the current density-voltage measurements were performed with EGaIn as the top electrode. The ferritin was tested only in the ±1 V sweeps range, and it showed a different behavior when compared to the encapsulin with a higher current density, especially when compared to EncH{} without cargo. Samples of the intermediate steps in the production of the Encapsulin SAMs were also prepared, which are the C_7S_2COOH and EDC-NHS activated lipoic acid SAMs. Both the SAMs show a significantly different behavior when compared to the encapsulin, with a higher current density for the lipoic acid SAM that is reduced by about one order of magnitude upon activation of the SAM. Differently from the encapsulin samples, the C_7S_2COOH and EDC-NHS activated lipoic acid SAMs were measured only at ±1.5 V sweeps range, as higher voltages resulted in breakdown of junctions causing short-circuits.

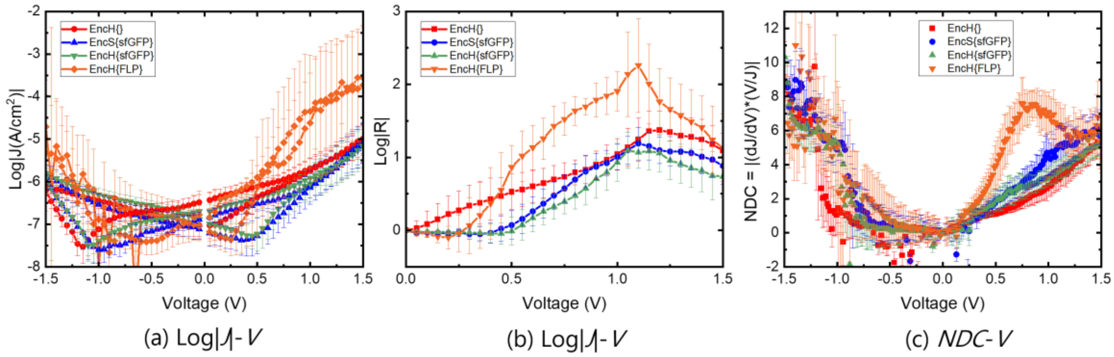

Figure S5. (a) Semilog plots of current density (<log|*J*|>$_G$) versus voltage (*V*) for the bias range of ±1.5 V for SAMs of EncH{} (red), EncS{sfGFP} (blue), EncH{sfGFP} (green) and EncH{FLP}

(orange). (b) Semilog plot of rectification (log|*R*|) versus *V*, and (c) plot of normalized differential conductance (*NDC*) versus *V*.

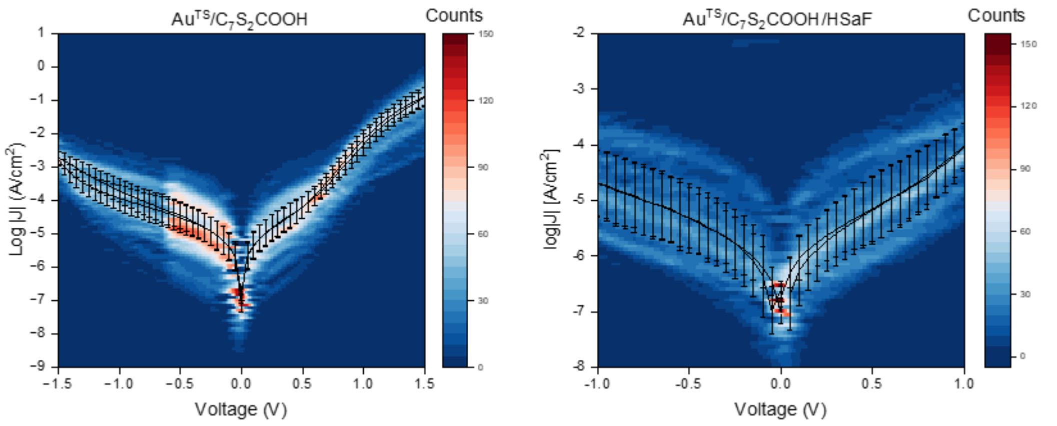

Figure S6. Heatmaps showing raw data of current density vs. *V* of Au/C_7S_2COOH//EGaIn and Au/C_7S_2COOH/HS-apoferritin//EGaIn junctions (HS-apoferritin here denoted as HSaF), overlapped with gaussian averages (black lines) with 95% confidence intervals as error bars.

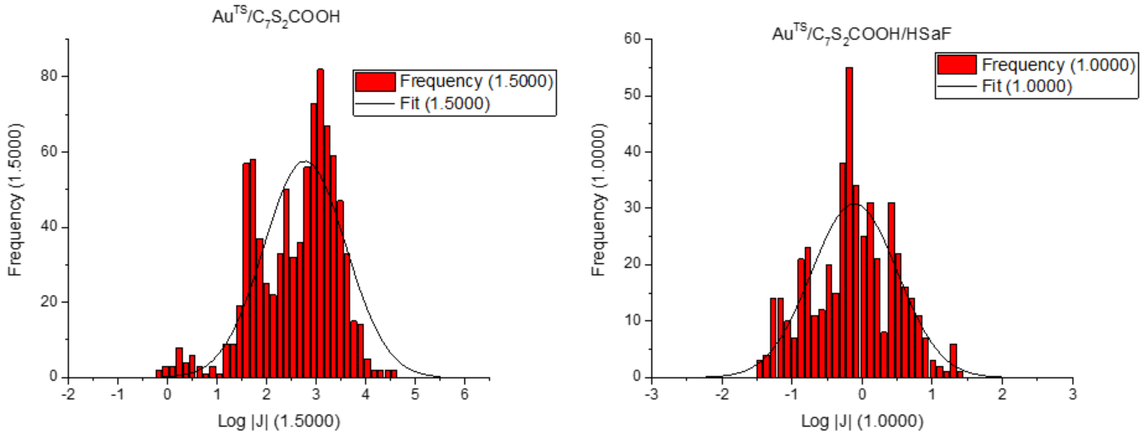

Figure S7. Current density histograms (in red) and their corresponding Gaussian fits (black line) of Au/C_7S_2COOH//EGaIn and Histograms of Au/C_7S_2COOH/HS-apoferritin//EGaIn junctions, HS-apoferritin here denoted as HSaF.

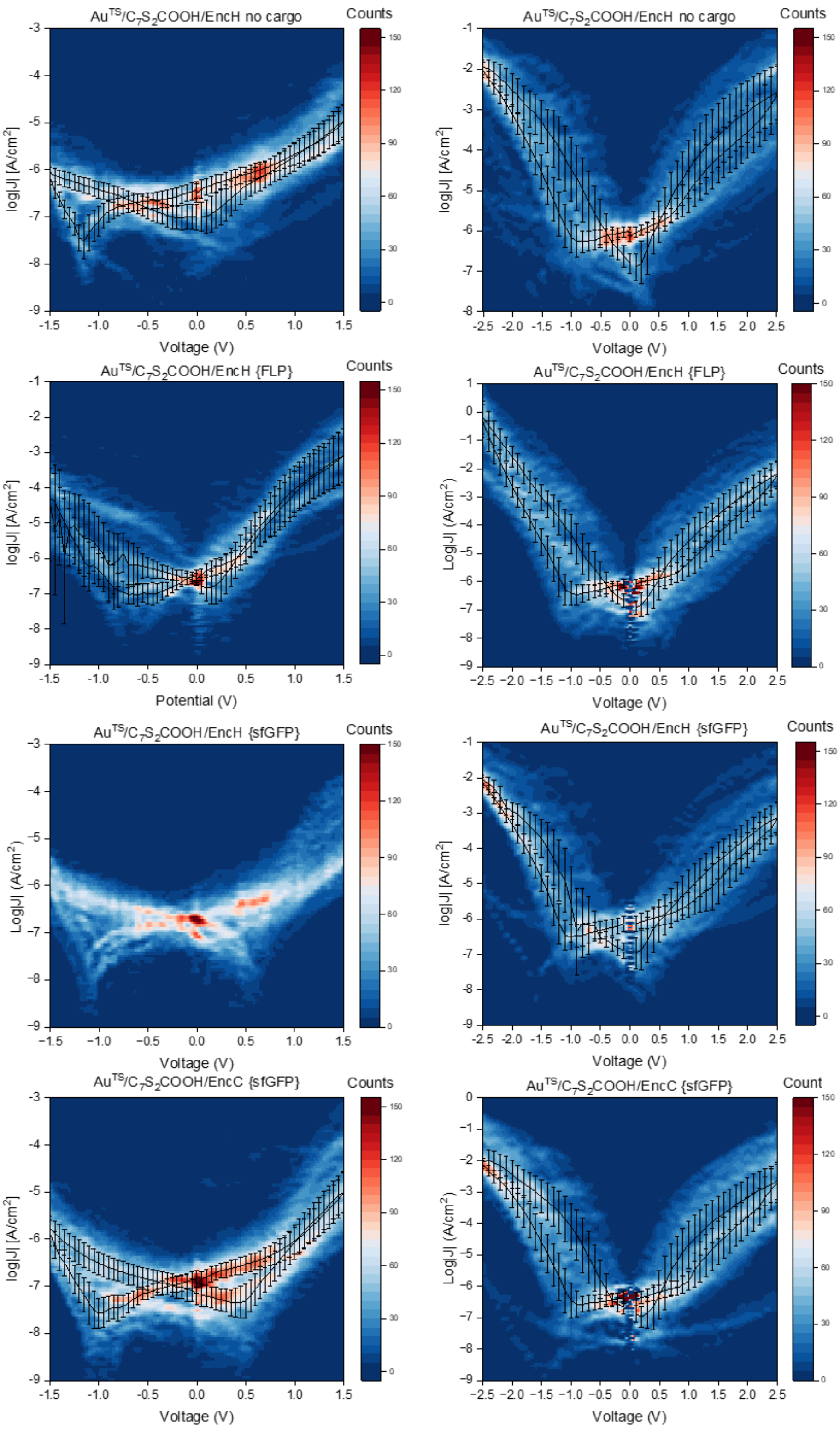

Figure S8. Heatmaps showing raw data of current density vs. voltage for the four encapsulin samples, overlapped with gaussian averages (black lines) with 95% confidence intervals as error bars.

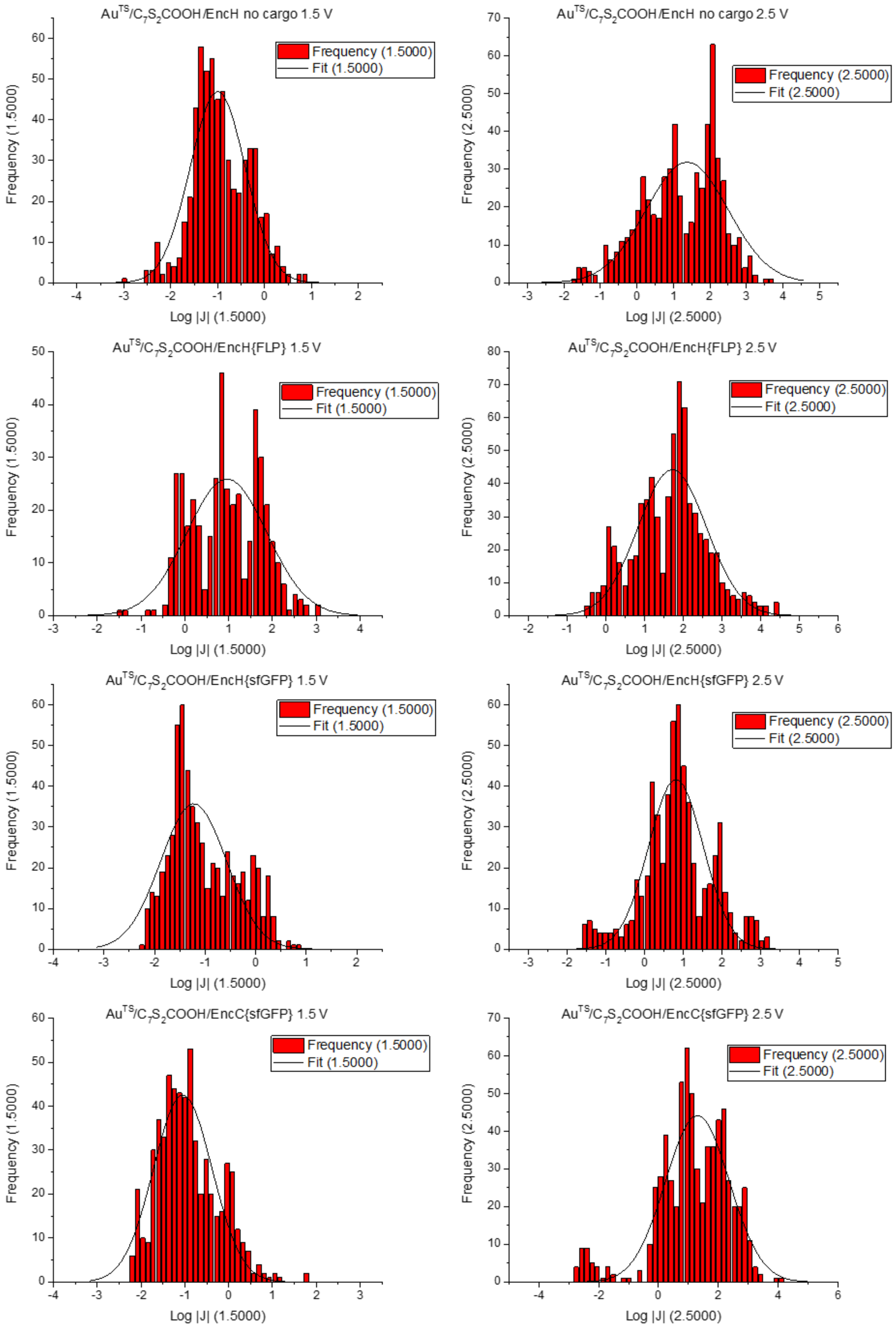

Figure S9. Current density histograms (in red) and their corresponding gaussian fits (black line) of Au/C_7S_2COOH/EncH{FLP}, Au/C_7S_2COOH/EncH{sfGFP}, Au/C_7S_2COOH/EncH no cargo and Au/C_7S_2COOH/EncC{sfGFP}.

References

[1] S. Michel-Souzy, N. M. Hamelmann, S. Zarzuela-Pura, J. M. J. Paulusse, and J. J. L. M. Cornelissen, "Introduction of Surface Loops as a Tool for Encapsulin Functionalization," *Biomacromolecules*, vol. 22, no. 12, pp. 5234–5242, Dec. 2021, doi: 10.1021/ACS.BIOMAC.1C01156.

[2] N. K. Gupta *et al.*, "Temperature-Dependent Coherent Tunneling across Graphene–Ferritin Biomolecular Junctions," *ACS Appl Mater Interfaces*, vol. 14, no. 39, pp. 44665–44675, Oct. 2022, doi: 10.1021/acsami.2c11263.

[3] N. K. Gupta *et al.*, "Switching of the mechanism of charge transport induced by phase transitions in tunnel junctions with large biomolecular cages," *J Mater Chem C Mater*, vol. 9, no. 33, pp. 10768–10776, 2021, doi: 10.1039/D0TC05773H.

[4] K. S. Kumar, R. R. Pasula, S. Lim, and C. A. Nijhuis, "Long-Range Tunneling Processes across Ferritin-Based Junctions," *Advanced Materials*, vol. 28, no. 9, pp. 1824–1830, Mar. 2016, doi: 10.1002/adma.201504402.

[5] E. A. Weiss, G. K. Kaufman, J. K. Kriebel, Z. Li, R. Schalek, and G. M. Whitesides, "Si/SiO2-templated formation of ultraflat metal surfaces on glass, polymer, and solder supports: Their use as substrates for self-assembled monolayers," *Langmuir*, vol. 23, no. 19, pp. 9686–9694, Sep. 2007, doi: 10.1021/LA701919R.

[6] X. Qiu *et al.*, "Thiol-free self-assembled oligoethylene glycols enable robust air-stable molecular electronics," *Nat Mater*, vol. 19, no. 3, pp. 330–337, Mar. 2020, doi: 10.1038/s41563-019-0587-x.

[7] N. Kumar Gupta *et al.*, "The Role of Structural Order in the Mechanism of Charge Transport across Tunnel Junctions with Various Iron-Storing Proteins," *Small*, vol. 18, no. 42, p. 2203338, Oct. 2022, doi: 10.1002/SMLL.202203338.
